# The Capitation Grant for Unallotted Persons

**Published:** 1913-11-22

**Authors:** 


### THE NON-PANEL MOVEMENT.

### The Capitation Grant for Unallotted Persons.

The difficulties confronting the administrators of the Insurance Act are indeed formidable. The general refusal of choice of doctor, now the cause of most of the medical difficulty, is no doubt being enforced against the better judgment of Insurance Committees all over the country; but the despotic rule of the Commissioners prevails, and it is indeed true that to concede free choice of doctor would mean not only the breaking of faith with the panel doctors but the collapse of the panel system altogether.

This difficulty can hardly have been foreseen when the Chancellor promised free choice of doctor in these words: "I am on all grounds in favour of a free choice of doctor. I think one of the essentials of curing is that the patient should have faith in his doctor; and you cannot have faith in your doctor if you have doctors thrust on you whom you have not chosen, who for various reasons, most of them unsatisfactory, the patient may not believe in" (the *Times*, June 2, 1911). The difficulty cannot have been foreseen when the official pink ticket was issued (though since withdrawn) with the words: "If you are arranging with the Insurance Committee to obtain your treatment from a doctor not on the list, and wish to claim a contribution towards the cost of the treatment, you must send this ticket to the Insurance Committee." The truth appears to be that it was assumed by Mr. Lloyd George that the remuneration offered would be sufficient to secure "the whole-hearted co-operation of the medical profession," to use his own words, without which it was admitted the Act must fail in respect of the medical service. He now finds that he must limit the promised free choice to the doctors on the panel lists, but that these lists are inadequate. In this predicament a fresh attempt is about to be made to attract additional men to the medical panels, or at least to maintain the present strength of the lists.

### The New Regulations.

With this object the Commissioners have issued fresh regulations, which will come into force in January next. Under these rules the local Insurance Committees will be relieved of the impracticable task of allocating by name to the panel doctors those insured persons who have not selected a panel doctor, but there will be an allocation by numbers. The panel doctor will receive capitation fees not only for the patients actually on his list, but in addition for a certain number of unknown persons, being insured persons who have the right to treatment under the Act and who may or may not exercise that right and apply to him for their treatment. This is an ingenious plan and will no doubt effect some improvement in the finances of the panel doctor. For the dislike of panel doctoring is so evident amongst the insured that nearly all wishing to take advantage of it have already made their selection. The others are paying private doctors out of their own pockets, or have decided to do so should they require medical attendance. In other words, the panel doctors will receive payment for a great deal of work they will never do. This arrangement has given dissatisfaction in quarters where it is alleged that the panel doctors are already overpaid considering the quality of their work, and the point has not been lost sight of by the Government's supporters in the press. Thus: "It can hardly be doubted that the insured persons who did not choose panel doctors, employed and paid doctors independently of the Insurance Act. This being so, the Insurance Commissioners are authorising the Committees to pay the medical profession twice for the same work" (*Daily News*, November 13). The newspaper quoted has shown of late a very decided change of front in its attitude towards the medical arrangements under the Act. Formerly all was "working smoothly," to quote the usual formula. Now we read (November 13): "It is some consolation that the present medical arrangements are only temporary, but that will be no consolation unless there is a determined effort to sweep them away at the earliest practicable opportunity, and to replace them with a State medical service which will bear comparison with the national educational service."

And a few days ago (November 1) we had: "The service is grossly inadequate, shamefully inadequate, considering the price that is paid for it. The public cannot permanently pay so heavily for so little, and it is time that serious attention was given to the reconstruction of the service." There are the weightiest reasons for doubting if public opinion will be ready for many years to come for the institution of a State service worthy of the name, and there seems little ground for anticipating any great change for the better if, and when, the present medical service is reorganised. It may be recalled that, prior to the Act coming into force, Dr. Addison, M.P., who may be regarded as the medical mouthpiece of the Government, wrote that to draw up the regulations for the medical benefits had been a task of the utmost difficulty and complexity, and it is not easy to see why a second effort should prove a notable advance on the first, even granting that the early working of the Act has disclosed the main points of weakness. It would, indeed, not be surprising if the problem of securing proper medical attendance for fifteen millions of people should prove so difficult of solution that the course finding most favour should be nothing less than a repeal of the medical benefits clauses of the Act. Be this as it may, it is certain that any substantial change in the present mode of administration must involve a resort to the melting-pot for the whole medical problem if it is to benefit either the insured public or the profession.

The non-panel movement, then, would seem to have full justification in the present unsatisfactory panel service, whose defects are now proclaimed even by those who till recently refused to admit them. Large numbers of the doctors engaged in working the Act are in hearty disagreement with the conditions of service; they are in a kind of unstable equilibrium, and very little would suffice to induce them to leave the panels. The powers that be are fully aware of the facts, and have clearly arranged, as above explained, for some advance in remuneration with the express object of limiting secessions from the panels.

### The German Doctors' Revolt.

As has already been mentioned in a previous article, the German medical profession is profoundly dissatisfied with the conditions of service under the German Insurance Law, which are, in many respects, inferior to those in this country. So much is this the case that a serious revolt is threatened, and a strike of doctors is being talked of for the New Year. Without entering for the moment into the merits of these disputes, or the causes of the unfavourable position of the profession in Germany, the extraordinarily disingenuous attitude of the *Westminster Gazette* is noteworthy —the one Ministerial daily newspaper with any pretensions to breadth of view and reasonableness. "The fact that our panel system is working so smoothly after one year may well arouse the envy and astonishment of the German public"; so says the *Westminster Gazette*. It can hardly have escaped the cognisance of the editor that, in the first place, "the envy and astonishment of the German public" not only do not exist, but are not even alleged to exist in his own paragraph: they are purely the figment of his contributor's imagination. Secondly, that the smooth working of the panel here is moonshine, and is being admitted to be so even by politicians and party newspapers on the Government side. And, thirdly, that to quote the miserable position of the German doctors as a "refreshing" proof that there is nothing "exceptional" in the vexations of insurance practice in Britain is a poor excuse indeed for the crude, hasty, ill-digested legislation of 1911, the true cause of real hardship and justifiable dissatisfaction not only to the medical profession, but also to millions of the insured in this kingdom.

